# Resident perceptions of Competency-Based Medical Education

**DOI:** 10.36834/cmej.67958

**Published:** 2020-09-23

**Authors:** Steve Mann, Amber Hastings Truelove, Theresa Beesley, Stella Howden, Rylan Egan

**Affiliations:** 1 Department of Surgery, Queen’s University, Ontario, Canada; 2 Faculty of Health Sciences, Queen’s University, Ontario, Canada; 3 Office of Accreditation and Education Quality Improvement, Faculty of Medicine, McGill University, Quebec, Canada; 4 Centre for Medical Education, University of Dundee, Scotland, United Kingdom; 5 Health Quality Programs, Queen’s University, Ontario, Canada

## Abstract

**Background:**

Residency training programs in Canada are undergoing a mandated transition to competency-based medical education (CBME). There is limited literature regarding resident perspectives on CBME. As upper year residents act as mentors and assessors for incoming cohorts, and are themselves key stakeholders in this educational transition, it is important to understand how they view CBME. We examined how residents who are not currently enrolled in a competency-based program view that method of training, and what they perceive as potential advantages, disadvantages, and considerations regarding its implementation.

**Methods:**

Sixteen residents volunteered to participate in individual semi-structured interviews, with questions focussing on participants’ knowledge of CBME and its implementation. We used a grounded theory approach to develop explanations of how residents perceive CBME.

**Results:**

Residents anticipated improved assessment and feedback, earlier identification of residents experiencing difficulties in training, and greater flexibility to pursue self-identified educational needs. Disadvantages included logistical issues surrounding CBME implementation, ability of attending physicians to deliver CBME-appropriate feedback, and the possibility of assessment fatigue. Clear, detailed communication and channels for resident feedback were key considerations regarding implementation.

**Conclusions:**

Resident views align with educational experts regarding the practical challenges of implementation. Expectations of improved assessment and feedback highlight the need for both residents and attending physicians to be equipped in these domains. Consequently, faculty development and clear communication will be crucial aspects of successful transitioning to CBME.

## Abstract (fr)

**Contexte:**

Les programmes de résidence canadiens effectuent un passage obligatoire vers la formation médicale fondée sur les compétences (FMFC). Peu de littérature documente les perspectives des résidents sur la FMFC. Comme les résidents senior agissent comme mentors et évaluateurs pour les résidents qui débutent, et qu’ils sont eux-mêmes des participants clés dans cette transition éducative, il est important de comprendre comment ils perçoivent la FMFC. Nous avons examiné comment les résidents qui ne sont pas actuellement inscrits dans un programme fondé sur les compétences perçoivent cette méthode de formation et ce qu’ils perçoivent comme avantages et désavantages potentiels, ainsi que leurs réflexions concernant sa mise en œuvre.

**Méthodes:**

Seize résidents se sont portés volontaires pour participer à des entrevues individuelles semi-structurées, avec des questions ciblant les connaissances des participants relativement à la FMFC et à sa mise en œuvre. Nous avons eu recours à l’approche de la théorisation ancrée pour élaborer des explications sur la manière dont les résidents perçoivent la FMFC.

**Résultats:**

Les résidents s’attendaient à une amélioration de l’évaluation et de la rétroaction, à un repérage plus précoce des résidents éprouvant des difficultés dans leur formation, ainsi qu’à une plus grande souplesse pour ajuster la formation selon les besoins d’apprentissages auto-identifiés par les résidents. Les désavantages comprenaient des problèmes logistiques entourant la mise en œuvre de la FMFC, la capacité des médecins traitants à fournir une rétroaction appropriée axée sur les compétences, ainsi que la possibilité d’une lassitude à l’égard des évaluations. Une communication claire et détaillée ainsi que des canaux pour la rétroaction aux résidents étaient des considérations clés à propos de la mise en œuvre.

**Conclusions:**

Les opinions des résidents concordent avec celles des experts en éducation au sujet des défis pratiques de la mise en œuvre. Les attentes en ce qui a trait à une amélioration de l’évaluation et de la rétroaction soulignent le besoin, autant pour les résidents que pour les médecins traitants, d’être bien préparés dans ces domaines. Par conséquent, la formation professorale et une communication claire représenteront des aspects essentiels d’une transition réussie à la FMFC.

## Introduction

*“The goal of all graduate medical education is to ensure that the graduating physician is competent to practise in his or her chosen field of medicine.”* DM Long^1^

Not many would contradict the essence of this statement; competent physicians are unquestionably the desired product of medical training. Questions, however, do present themselves: Do historical understandings of “residency” or “postgraduate training” remain applicable to modern medicine, with its exponential growth of information and technology? What, exactly, is a competent physician, and who (or what) determines, and measures, competence?

Many would argue that successful completion of a predetermined number of years in training does not guarantee competence to practice medicine independently.^2^^-^^6^ In addition, legislated duty-hour restrictions have recently curtailed the number of hours that postgraduate medical trainees spend engaged in clinical contact, prompting concerns that training programs no longer provide sufficient exposure to ensure competence.^7^^,^^8^ North American licensing bodies such as the Royal College of Physicians and Surgeons of Canada (RCPSC, the “Royal College”) historically have depended on completion of a time-based residency training program, successful sitting of specialty examinations, and the attestation of residency program directors.^1^ Despite successful attainment of these criteria, however, both trainees and program directors continue to have reservations about competence upon graduation.^9^

In response to these concerns, competency-based medical education (CBME) proposes to focus on learners’ abilities as the intended product of education, rather than on the instructional process.^2^^,^^10^^-^^15^ The transition to CBME (termed “Competence by Design” or CBD by the Royal College) has been mandated across Canada. This transition has a rolling timetable of implementation by training speciality between 2017 and 2022.^16^

Much of the literature concerning competency-based training is written from the perspective of educators and administrators who are involved in designing, planning, and overseeing the implementation of CBME. However, despite calls to include learners in this discussion, we found few published studies that examine residents’ perspectives. The resident voice has been conspicuously absent from discussions regarding the impact of transitioning to CBME, even though trainees are most directly affected by this change.^17^^-^^20^ Because upper-year residents act as mentors and assessors for incoming cohorts, it is important to understand how they view CBME. Their perspectives may also help inform the approach to CBME implementation taken by administrators, educational leaders, and clinical faculty.

When Queen’s University in Kingston, Ontario, announced that all incoming residents would enter CBME programs in July 2017, CBME curricula for PGY1s were intended to run concurrently with existing training for the upper-year residents who continued to move through a traditional time-based medical education curriculum. Current residents were not expected to experience disruptions in their studies because of Queen’s transition to CBME. However, with this significant curricular innovation being implemented around them, non-CBME residents would have to engage with these changes as assessors of, and mentors to, incoming residents. In this respect, understanding residents’ perspectives is as important as understanding faculty perspectives about CBME. We also felt it was important to understand the perspectives of non-CBME residents in the training years immediately preceding this innovation, particularly as these residents would be engaged to pilot some aspects of CBME assessments in their departments in advance of the inaugural CBME cohort.

We conducted a series of interviews to identify non-CBME residents’ perceptions of CBME, of running two learning streams (CBME and time-based concurrently), and their understanding of the benefits and challenges associated with this transition.

## Methods

We conducted this study at Queen’s University, a medium-sized institution in Kingston, Ontario. Since Queen’s was scheduled to transition all Royal College training specialties to CBME in July 2017, shortly after this study was conducted, residents were superficially familiar with the notion of CBME, but did not have first-hand experience. Via email, we invited all Royal College specialty and subspecialty residents at Queen’s University (approximately 400) to participate in this study. Family medicine residents were not included, as this residency program had already made the transition to a competency-based curriculum. Participants received an information letter with the recruitment email and completed a consent form at the beginning of their interview sessions.

We employed a social constructivist viewpoint, as articulated by Charmaz.^24^ This emphasises knowledge creation based on diversity and the complexities of views and actions. Given the lack of existing data regarding resident perspectives of CBME, we felt that a grounded theory approach was appropriate as we were interested in understanding resident perceptions of this new teaching method and how they felt it would impact training. In particular, Boeije describes the constant comparative method, whereby comparisons are made within and between single interviews and groups in order to develop categories, define concepts, and identify patterns, as well as refine the interview process over time.^25^ This informed a research-aligned design of the interview process, data coding, and analysis.

Based on this desire to understand the complexities of residents’ perceptions, we felt that individual semi-structured interviews were most appropriate. Compared to the more restricted responses possible by survey, we felt these allowed exploration of individual expectations, concerns, and opportunities, whilst maintaining the ability for redirection if the discussion moved off-topic.^23^ We also considered focus groups, but decided that the scheduling difficulties would be impractical.

One of the authors (AH) carried out all interviews, which were audio recorded, transcribed, and de-identified, with only sex, year of training, and program being retained within the transcript. To identify participants’ baseline knowledge about CBME, at the start of each interview we asked them to describe their understanding of CBME, regardless of how accurate they felt that understanding to be. The interviewer then read a brief script outlining the RCPSC CBME implementation guidelines (see Appendix A). This script provided all participants with a standardised understanding of CBME to direct their comments but was carefully screened to ensure that the language and/or positioning of CBME was as unbiased as possible in order to minimise the effects of our own perceptions of CBME as educators, administrators, clinical faculty, and recent residents. Question topics included perceived personal, specialty program, and institutional advantages and disadvantages of CBME. We developed questions by means of collaboration between the lead author (SM), who is a clinician and medical educator with expertise and interest in CBME, and two researchers (RE and AH) with expertise in qualitative methods and backgrounds in educational research. We asked residents to name the greatest potential advantage of CBME as well as the greatest challenge, and finally to provide their thoughts on what might make the transition to CBME easier. Interviews lasted from 35 to 60 minutes (average 43 minutes).

Coding consisted of open coding (developing of categories and labels) and axial coding (conceptualising the patterns and differences between groups). After analysis of the first three interviews, we refined the interview guide, and added questions regarding the greatest perceived advantage and disadvantage of CBME. Axial coding failed to demonstrate differences between medical and surgical residents, and we treated all participants as members of the same group. We refined the themes and categories identified during open coding. and halted recruitment with the advent of CBME for all incoming residents of July 2017 and based on analysis demonstrating that saturation had been reached.

Coding was conducted using qualitative analysis software. Two coders (AH and SM) individually coded the same transcripts. Discussion of their coding allowed for refinement and agreement of codes, and integration and code book development by researchers with backgrounds in medical education and qualitative research. Once interrater reliability had been achieved, AH conducted the coding for the remaining transcripts. Any language or repetition of perspectives or comments provided in the script were identified, and unless contextualized within the respondent’s context, were dismissed.

The three researchers (AH, SM, and RE) have extensive experience with CBME. This knowledge comes from lived experience (SM), and from administrative program development and research (AH and RE). The researchers support CBME but also believe that implementation must be collaborative and based on a diversity of perspectives and needs. Memoing and reflexive annotations were used to identify *a priori* positions held during coding, and we sought as much as possible to identify and consider potential hidden biases through conversation.

The institutional Research Ethics Board approved this study (REB 6015347). Participants received a $15 Starbucks gift card.

## Results

Of the approximately 400 residents who were invited, sixteen participated in interviews (see Table 1).

**Table 1:** Participant characteristics

Characteristic	Participants (total 16)
Sex	
Female	8
Male	8
Year of Training	
1	5
2	5
3	4
4	2
Training Program	
Internal Medicine (IM)	7
Physical Medicine & Rehabilitation (PMR)	1
Obstetrics & Gynaecology (OBGYN)	2
Emergency Medicine (EM)	1
Orthopaedic Surgery (OS)	3
General Surgery (GS)	1
Critical Care (CC)	1

We conducted interviews from November 2016 to April 2017. Over the six-month interview period, participants demonstrated increasing awareness of how CBME might affect their own programs, as evidenced by statements about discussions with their program directors or academic advisors. The volume and specificity of information on CBME provided by individual programs varied between specialties, and there is no detailed information available as to what information was communicated by each training program to its residents at particular time points.

The topics of assessment and feedback, teaching and learning, and the details of implementation emerged repeatedly, and the developed categories were distributed amongst four themes: rumours, perceptions of assessment and feedback; perceptions of teaching and learning; and implementation of CBME. Residents from surgical and medical specialities and different years of training reported similar perceptions of CBME; therefore, data from all participants are presented together. We used participant number, year of training, and program as identifiers for participants’ quotations.

### Rumours contribute to resident perceptions of CBME

This introductory theme may be considered all-encompassing, in that rumours contributed to all perceptions and views of CBME. Rumours were not the sole source of information upon which residents based their perceptions of CBME; they also referred to information received from Queen’s University or from their own training programs. Interestingly, however, no participant mentioned seeking information from the Royal College website. The first question of the interview, “What do you currently know about CBME?” frequently resulted in residents alluding to hearsay from friends and colleagues, perhaps in other training programs or at other universities, who had either already experienced CBME first-hand or had heard information about it. Those participants who were interviewed closer to July 2017, or who were more involved in their program’s CBME design, often expressed views with greater certainty, but even the best-informed residents expressed uncertainty about the details of CBME implementation. Potential advantages, along with perceived disadvantages, are discussed within the ensuing themes.

### Perceptions of assessment and feedback in CBME

The theme of assessment and feedback occurred repeatedly during all interviews, accounting in some fashion for the majority both of concerns and positive expectations (see Table 2). Feedback was closely linked to assessment, whether formative or summative, and the terms feedback and assessment were used interchangeably by participants, who expected all teaching-learning encounters and feedback opportunities to be associated with formal documentation and assessment. Residents anticipated that implementation of CBME would improve feedback as it would require feedback mechanisms that are superior to those that were in place. They also expected changes to assessment culture that would make asking for and receiving feedback more commonplace. Conversely, they identified the additional time required for both residents and attending physicians to complete assessments as a significant concern. Whilst residents anticipated that CBME would provide a greater assurance of competence, they also identified that a clearer definition of competence was required.

**Table 2:** Assessment and feedback subthemes

Subtheme	Description	Representative quotation
**Improved Feedback**	CBME will require feedback mechanisms superior to those currently in place. Frequency, timeliness, and specificity were feedback qualities which residents anticipated would be improved by the implementation of CBME.	Certainly more feedback…and not only more, but more specific, so you know exactly where your weaknesses might be and where you need to develop skills. I see that being one of the biggest advantages [of CBME]. (P4, R2 OBGYN)
**Change in feedback culture**	Residents expected CBME to change the expectations surrounding feedback, such that asking for it, or receiving it even without asking, would become more expected and commonplace.	Part of my understanding with CBME is that with closer assessments, that attending physicians are almost expected to then observe us on a more frequent basis. And although I think it is quite good to be observed and know what I am doing that is right or wrong, it will require a change in culture. (P7, R3, IM)
**Defining competence**	Residents expressed a belief that it was possible to progress through current residency training and licensure without necessarily being competent. They felt that CBME would provide greater assurance of competence by means of increased volume and quality of assessment, although there was concern that a practical definition of competence is lacking.	[The main benefit of CBME is] to know that they [the graduating resident] were not the person who just got through because someone was not looking or they got lucky. They did not make it through residency without getting all the skills and knowledge that they need, because those things have been evaluated over and over and over again. They have been declared to be competent. (P11, R2 OS)
**Time requirements**	A more robust mechanism of assessment and feedback will require increased time from both residents and attending physicians. This was frequently mentioned as one of the most significant challenges raised by CBME.	I think the biggest challenge is going to be just the amount of time and energy more from the staff than from the actual residents themselves to ensure that [feedback occurs]. Now they have to be hands on. There is no avoiding every single day or every single period they need to go through specific goals. (P15, R4 OS)

### Perceptions of teaching and learning in CBME

Resident perceptions of teaching and learning centred around clear objectives and responsibilities. Despite their concerns that difficulty defining competence and increased assessment burden might interfere with achieving the goal of improved feedback, participants felt very strongly that CBME would lead to both increased clarity of learning objectives and greater impetus for residents to be more self-directed in their learning.

Although the differences between objectives, expectations, and competencies are explored in detail in medical education literature (see, for example, Frank et al^13^ and Harden^26^), participants used these three terms interchangeably both within and between interviews. Referring to the concept of what residents were expected to know and to do, all three terms were used to express standards of clinical knowledge and performance. Residents expressed an expectation that CBME implementation would result in clinical services providing rotation-specific learning objectives or competencies which were detailed, clear, and explicit.

The biggest [advantage of CBME] is that the expectations are very clear. Right now, you only figure out what the expectations are when you have done something wrong. So, I think that…knowing exactly what you need to do in order to be successful in each required step and having it all laid out when you start is very nice and very clear. (P11, R2 OS)

Residents linked clear learning objectives to improved performance in three ways. First, the improved feedback which was expected to result from CBME meant that they would receive specific, timely, and detailed feedback which was aligned with expected competencies, allowing them to identify areas in which they were achieving these, and where further development was needed. Secondly, they anticipated receiving more directed, objective-based teaching by attending staff. Thirdly, residents stated that clear objectives and expected competencies for a given stage of training would give them a better capacity to accurately gauge their own performance and progress.

This capacity for reflection was linked to an expectation that residents would need to take a greater degree of ownership of and responsibility for addressing their own learning needs with a CBME approach. This was most commonly expressed in terms of self-reflection, whereby residents would be better able to identify for themselves gaps in their knowledge and understanding.

I am a student and I like to own my own learning and like to be able to identify what I know and what I don’t know. I think the biggest problem [with current training] is sometimes we don’t know what we don’t know. I think by having this format [CBME objectives] put out we will be able to identify...I will finally be able to identity and see clearly, ‘oh shoot it is this that I don’t understand.’ (P7, R3 IM)

*Resi*dents also expressed an expectation that CBME would provide greater latitude for self-directed learning, allowing residents to pursue opportunities to address these knowledge gaps outside of the relatively constrained framework of conventional training.

So, I think an advantage [of being responsible to achieve competencies] is that you can try to actively seek out opportunities that you might not be aware of otherwise…you might be inclined to go above and beyond to try to seek out those opportunities. (P5, R3 OBGYN)

This expectation of self-directedness encompassed both the notion of residents identifying their own learning needs by means of clear expectations, and of having the freedom to engage in additional or more relevant learning opportunities because of greater flexibility resulting from CBME.

### Implementation of CBME

Many participants expressed uncertainty or even pessimism regarding the more practical implications of the transition to CBME but did suggest some strategies by which these difficulties might be mitigated (Table 3). Logistical challenges were the most commonly identified concern, and residents also questioned whether CBME would result in truly meaningful educational changes. Communication and opportunities for resident input and feedback throughout the implementation process were repeatedly suggested as vital to a successful transition to CBME.

**Table 3:** Implementation subthemes

Subtheme	Description	Representative quotation
**Logistical challenges**	Concerns centred around the availability of educational, technological, and administrative support systems for the expected increase in workload. This workload involves both increased assessment volume, and logistical challenges which CBME might entail in terms of scheduling residents who might progress through training at different rates.	It would be a scheduling nightmare. I can’t imagine what that would be like for the administrators. I pity them because I think they already have a challenge scheduling people on rotations and trying to make sure that calls are covered and that services are adequately staffed and stuff. And so if you had people accelerating through you may end up with only half the number of R1’s that you thought you were going to have. (P9, R1 IM)
**Theory vs practice**	Despite expectations of higher-quality feedback and clearer objectives, many participants questioned whether the theory of CBME would translate into tangible educational changes, and what would be the practical implications for their learning. If the transition to CBME simply involved a “re-branding” of existing training the potential for real positive change may be limited.	So competency based medical education sounds nice, but what are we doing now then? Are we not training people to competencies and how is it going to be fundamentally different? And how does that [CBME] change the structure of my day and my month and my block and stuff like that?... I still don’t feel like the details of what that actually means and how that is fundamentally different from what we are doing now are being communicated to the residents, other than it is more assessment. It has to be more than that. (P1, R1 IM)
**Recommendation for education and communication**	Participants focused on the details and logistics of this process, emphasising education and communication. Key points were open lines of communication to program directors and the postgraduate medical education office for residents to offer feedback and suggestions during the transition.	*I myself don’t have a lot of information on the process [of CBME implementation]. I think that leads to a lot of the questions and resistance. I think the easiest thing is to give people a heads up on what is going on but also more concrete information on how this is going to look. (P8, R2 IM)*
**Potential loss of positives**	Participants noted a strong bond of collegiality both within and between years of training, and even between programs. There was concern that transitioning to CBME would result in loss of this sense of togetherness and commonality of experience and goals and a “disconnect” between junior and senior residents.	*Part of why I came here is that you know all the residents really well and everyone is in it together and is really supportive of each other. If your six 4th and 5th years are going through totally different from your 1st, 2nd, and 3rd years then there might be a bit of a disconnect and not as great of an understanding of what the other group is going through. (P4, R2 OBGYN)*

## Discussion

These observations describe what residents expect to be the potential advantages, disadvantages, and considerations surrounding the upcoming transition to CBME.

### Advantages

Participants expected that many of the primary advantages of CBME would occur with respect to improved assessment and feedback. Interestingly, in anticipating this as a benefit of CBME, residents viewed feedback from a predominantly passive perspective. With the exception of an expectation that they would bear more responsibility for “getting forms filled out,” participants did not suggest a more active role for themselves in this process, concentrating on the informational aspect of feedback from an assessment standpoint, rather than on their own reactions to feedback or the cyclical process of improvement.^27^ Although residents suggested that CBME would allow for more self-directed learning with respect to identified knowledge gaps, the concepts of self-reflection and analysis specifically with respect to feedback were lacking in their responses. This suggests that residents may lack insight into their own roles in improving and enriching the process.^28^

An improved ability to identify and support struggling trainees was a second potential advantage of CBME. Describing the current practice as a tendency to “pass along” struggling or borderline residents to the next clinical rotation, residents highlighted current limitations in receiving constructive feedback. Reddy et al identified lack of comfort amongst attending physicians in providing constructive feedback as a major barrier to effective feedback processes, and recommended ongoing faculty development, as well as changes in the culture of feedback that prioritize “coaching” rather than “judgement.”^29^ Participants in this study also reported their hope that a culture change would accompany the implementation of CBME. The optimal implementation of CBME would result in requesting and receiving both positive and constructive feedback more routinely, and consequently more easily. The actual means by such a change would occur were not explored; it seemed, somewhat optimistically, to be an expected part of the implementation process.

Discussing the definition of competence, participants expected a lower likelihood of a struggling trainee “slipping through the cracks” and passing the Royal College licensing examination despite a lack of true clinical competence. Existing literature suggests that a significant minority of graduating residents may be, or at least feel, unprepared for independent clinical practice.^1^^,^^9^^,^^30^^,^^31^ This is one of the potential consequences of an assessment culture which “fails to fail,” but it is not yet clear whether struggling trainees in a CBME environment will substantially improve or be allowed to “fail.”^32^

A third perceived advantage of CBME was potential flexibility in training time. Although not all participants felt that training periods would be shortened, most did expect a greater degree of flexibility within clinical rotations. This was related to their expectations that clearly outlined objectives or competencies, combined with improved feedback, would allow residents to more effectively reflect on their own progress, identify areas in which knowledge or skills were lacking and seek out opportunities to address these areas with greater independence. This reflects residents’ grasp of one of the fundamental goals of CBME as designed by the Royal College, which is to help residents identify and address their own learning needs as they develop the necessary skills to be life-long learners.

### Disadvantages

Perceived disadvantages and challenges were predominantly of the practical or logistical type, as described by Hawkins et al, with residents not expressing many of the conceptual or theoretical challenges which have been identified by educators.^33^

The greatest perceived disadvantages of CBME were the logistical challenges of scheduling and time requirements for increased assessment. Residents had a realistic view of the administrative difficulties which even moderately flexible clinical rotations would entail and expressed uncertainly as to how these could be overcome within the framework of training programs which had to balance educational needs with patient care responsibilities. Suggesting that more resources would have to be committed to patient care in the form of additional physicians or physician assistants, residents questioned whether such supplementation would be feasible within the fiscal constraints of the Canadian health care system.

Most residents acknowledged that a “pure” CBME model, in which trainees moved from rotation to rotation as soon as the requisite competencies were achieved, would not be feasible without additional clinical support to ensure adequate ongoing patient care. Whilst some envisioned this as the ultimate goal of CBME, most anticipated training which remained fixed within scheduled clinical rotations whilst incorporating a greater degree of flexibility or freedom within those rotations. This accords with the Royal College’s own stated outline for CBME, although no participant made reference to material from the Royal College or information from its website.^16^

Participants described the second primary disadvantage of CBME as the potential for assessment burden and fatigue. Many residents felt that the time needed for them to take ownership of their learning and seek regular assessments from attending physicians would have to be found in addition to their already busy clinical and academic schedules. Several also noted that if there were a formal expectation for documented daily assessment and feedback activities, these could become a burden and likely a matter of rote. These concerns were also expressed by residents interviewed by Ross et al, who felt that the pilot competency-based program which they experienced led to “over-assessment.”^20^ This risk of competency-based programs devolving into an endless series of tick boxes has been identified previously.^34^

Residents also expressed uncertainty regarding faculty views of CBME. They questioned attending physicians’ support of CBME in relation to the additional time commitment required, as well as attendings’ ability to deliver the quality of feedback required by CBME. Similar to the views reported by Boet et al, residents felt that if faculty did not receive suitable training and development, it was possible that CBME would not change existing feedback in a meaningful way.^19^ Simply exchanging one assessment for others, in greater numbers, does not represent positive change. Given that attending physicians, the frontline educators in the clinical teaching environment, also have multiple competing interests and priorities for their limited time resources, the increased assessment burden and increased administrative responsibilities associated with CBME could have a deleterious effect on the amount of clinical teaching and genuine feedback available.

### Considerations surrounding implementation

Many of the considerations surrounding CBME implementation related to the details of what exactly was intended. From this perspective, residents were acutely aware of their lack of clear, precise information. Many of their perceptions and expectations were based, in whole or in part, upon rumours and second-hand information. Some participants who were interviewed within six months of CBME implementation had more specific information from their programs about what to expect from CBME, but this varied substantially between and even within programs, indicating that efforts on the part of training programs to educate their residents about the upcoming transition had met with mixed results.

In a similar fashion, many participants expressed uncertainty about what exactly constituted competence, and how it could be defined and assessed. In this, they are not alone. Educators, program directors, and residents have all commented upon the lack of clarity in defining this key term.^13^^,^^19^^,^^26^^,^^33^

In order to combat these uncertainties, residents advocated for clear communication. Acknowledging that the transition to CBME would be associated with some difficulties and disruption, they spoke to the need for clear information from their program directors in order to understand what was expected. Programs preparing to transition to CBME should communicate with their residents as clearly and promptly as possible throughout the process.

In addition, residents expressed a desire for the opportunity to provide feedback prior to and during the implementation process, feeling that rapid addressing of concerns or difficulties, and confirmation of positive functioning, would facilitate the transition to CBME. This is in accordance not only with their desire to be treated as adult learners, but with existing literature speaking to the benefits of involving residents in curriculum design and implementation.^11^^,^^35^^,^^36^

Finally, residents’ comments that feedback mechanisms often failed to meet their needs reflects a requirement for ongoing faculty development to ensure that attending physicians are able to provide the type of feedback expected within a CBME framework. This is echoed by Fraser et al, who identified a lack of competence amongst attending physicians with respect to the assessment and feedback required to successfully deliver CBME.^37^

## Strengths and limitations

We used a sampling frame in order to ensure that our participants represented as diverse a population as possible, including residents from a variety of specialties and years of training. Despite this, sixteen residents are unlikely to form a representative sample from a total population of approximately four hundred, or to adequately convey all potential viewpoints. The exploratory nature of this work means that further research is necessary. Although all residents were invited to participate, there is a risk that only those who were most interested in or informed about CBME chose to be interviewed, introducing bias and leading to under-representation of the perspectives of less-interested residents.

The extended timeline of this study, with interviews occurring over six months, was due to difficulty scheduling participants. Surgical residents in particular have very little free time, and despite the interview timetable being extremely flexible, this remained a challenge throughout the study period.

We carried out this study in accordance with recognised research principles, guided by other published studies with similar purposes, in order to answer the research question.^19^^,^^20^ Our findings therefore may serve to inform educators, administrators, and researchers at both the local and national or international levels. In fact, we have begun a Canada-wide survey-based study of all residents with questions informed by the themes developed during this project. It would also be valuable to conduct similar work in other countries which would help to confirm and refine this study’s wider applicability.

### Conclusion

Residents at Queen’s University anticipate many advantages associated with CBME, particularly with respect to improved assessment and feedback, increased clarity of learning objectives, and greater flexibility and self-directed learning. Perceived disadvantages include significant logistical challenges regarding implementation, tension between educational opportunities and responsibilities of patient care, and the potential for increased assessment burden on both residents and attending physicians. Residents advocated for clear communication regarding CBME implementation, particularly with respect to the practical details of rotation scheduling and definitions of competence, and open channels through which to provide their feedback on an ongoing basis. The Royal College and Canadian residency programs should actively engage with residents regarding CBME implementation and should provide training to both faculty and residents to help maximise effective feedback dialogue. Understanding residents’ expectations and apprehensions will allow training programs to tailor orientation activities and introductory rotations to ameliorate concerns and enhance the benefits of CBME.

## Appendix A CBME script

Now I would like to provide you with an official account of what CBME is, and how it is expected to influence residency training. This is to provide you some of the background to inform your answers as you consider upcoming questions.

As you may know, the Royal College of Physicians and Surgeons has mandated that all residency programs transition to CBME.

- Competency based medical education is an outcomes-based approach to the design, implementation, assessment, and evaluation of a medical education program using an organizing framework of competencies.
- Identify the outcomes first, then design the assessment and evaluations to be in alignment.
- Time based vs competency based

The framework for CBME comes from the Royal College of Physicians and surgeons CanMEDS framework which identifies and describes seven roles that lead to optimal health and health care outcomes.

- The expectation of the Royal College is that the milestones that are connected to the CanMEDS competencies will provide learners with a transparent path from novice to certification, and finally to becoming practicing physicians.

You will be able to clearly identify expectations, and the Milestones you are expected to meet, as well as strengths and areas that may need improvement. This will enable learners to identify their own individual needs and abilities at an earlier stage, and to obtain more timely support if additional learning needs activities are needed.

- The transition to CBME will require frequent assessment from faculty, both formal and informal, and the development of new assessment tools.
- The Royal College is adopting an approach to CBME implementation which will roll out each specialty over time, resulting in all programs starting by 2021.

Queen’s has chosen to fast track this approach, and is aiming to have CBME implementation across all specialty programs for the incoming cohort of 2017.

- I should note that Rather than change the program for residents mid-stream, the Royal College proposes running both methods of delivery concurrently. New cohorts will enter programs that use CBME, while current residents will continue in their existing time based streams.
- Naturally there are pros and cons with the implementation of any new programming, and we are interested in residents’ perspectives about these upcoming changes.

Do you have any questions about what I have just read? Is there any part that you would like me to repeat or further clarify?
